# Human faecal collection methods demonstrate a bias in microbiome composition by cell wall structure

**DOI:** 10.1038/s41598-019-53183-5

**Published:** 2019-11-14

**Authors:** Emma-Jane Watson, Jennifer Giles, Benjamin L. Scherer, Paul Blatchford

**Affiliations:** 1 CSIRO Health and Biosecurity, Gate 13 Kintore Avenue, Adelaide, South Australia 5000 Australia; 2 Zespri International Limited, 400 Maunganui Road, Mt Maunganui, 3149 New Zealand

**Keywords:** Applied microbiology, Microbial ecology

## Abstract

Clinical trial faecal collections present challenges through geographical spread and inexperienced participants. Collection techniques have been developed and tested to overcome these challenges, but previous studies investigating these techniques have demonstrated a highly variable capacity for sample preservation. Furthermore, these studies typically only examine either preservation of genetic content or metabolites, not both. This study investigated the Stool Nucleic Acid Collection and Preservation Tube (Norgen BioTek Corp) for the preservation of both microbial DNA and microbial organic acid metabolites in human faecal samples when compared to frozen samples. Twenty six healthy adult participants were instructed to collect a bowel movement, subsample into collection tubes and immediately transfer the remaining bulk to −20 °C storage. Resulting organic acid concentrations remained comparable across methods when the preservation tubes were used correctly. The 16S rRNA gene sequencing data revealed twenty significantly different bacterial genera between the two collection methods. Ten Gram-negative genera were more abundant in the collection tubes, and ten Gram-positive genera were more abundant in the fresh frozen samples. This study has illustrated that faecal collection methods bias the microbial community profile according to Gram status and this should be considered when designing studies that collect and store human faecal samples.

## Introduction

Growing interest in the colonic microbial community and its relationship to health have prompted increased investigation into human microbiota structure and function^1^. One of the more recognised areas of microbial metabolism is the production of organic and short chain fatty acid (SCFA) compounds^1,2^. The SCFA content of faecal samples is commonly measured with analyses such as gas and high pressure liquid chromatography^3^. The microbiome population can be investigated using a range of techniques including fluorescence *in situ* hybridization, quantitative PCR, 16S rRNA gene sequencing as well as culture-dependant methods^4,5^. These powerful investigative tools are routinely used for the analysis of faecal samples but rely on accurate and reproducible sample collection, storage and processing.

Commonly used faecal collection methods include: immersion in Tris-EDTA, RNAlater or ethanol, commercial stool preservation tubes, and immediate transfer to −20 °C (fresh frozen) or −80 °C storage^6^. Although there are many available options, large clinical trials present faecal collection challenges through large sample size, geographical spread, and self-collected samples from inexperienced participants. Previous methods used by our research laboratories include the use of transportable freezers and bulk sample collections. This method can be very laborious, time consuming and consequently, costly. Commercially available preservation tubes represent a simple approach that have previously shown promise for the preservation of microbial DNA. A recent study explored preservation capacity of the commercially available OMNIgene.GUT (DNA Genotek) collection tubes and used 16S rRNA sequencing to demonstrate minimal divergence from −80 °C frozen samples^7^. Another group also confirmed these findings when comparing −20 °C frozen and collection tube samples^8^, and a different study found no significant differences between the 16S rRNA sequencing profiles produced from the collection tube and fresh samples^9^.

A commercially available system that has been developed for faecal preservation is the Stool Nucleic Acid Collection and Preservation Tube (Norgen BioTek Corp, Cat. 45630)^10,11^. The Norgen tube has been compared directly to the OMNIgene.GUT tube and no significant difference was observed for either α- or β-diversity or phylum and genus level composition^12^. The Norgen tube has been used for DNA and RNA preservation in a range of human and animal trials^13–17^. Furthermore, the Norgen tube can hold a sample of up to 2 grams which is a considerable increase in sample size when compared to other commercially available collection tubes.

The present study aimed to define the applicability of Norgen preservation tubes (collection tubes) for the analysis of faecal microbiome and organic acid metabolites in a clinical trial setting, compared with the standard frozen method (fresh frozen). The Norgen tube was selected for use as it supported a larger sample size which allowed for both DNA extractions and metabolite analysis.

## Materials and Methods

### Participant recruitment

Healthy adult participants were recruited with written informed consent under CSIRO Human Research Ethics Committee approval number 09/2015. All methods used were conducted in accordance with the guidelines and regulations outlined by the ethics approval. We aimed to test a wide range of faecal samples across the collection methods so no exclusion or inclusion criteria were used during recruitment. Participants were asked to collect their entire bowel motion into a plastic toilet liner collection bag and then subsample into two collection tubes from different locations on the faecal sample. Participants were also asked to complete a Bristol Stool Scale questionnaire once they had completed their sampling. The remaining bulk sample in the liner bag was immediately transferred to −20 °C storage (fresh frozen), with cold chain storage of samples maintained at −20 °C using transportable freezers until received at the analytical laboratory. The tubes were sent at ambient temperature through standard Australian Post mailing system to our analytical laboratory. This study aimed to recreate a clinical trial setting and so depending on participant geographical location the tubes spent varying times in transit. However, all tubes were received at the analytical laboratory within six days post collection. When received at the laboratory, the fresh frozen and collection tube samples were transferred to −80 °C storage. Fresh frozen and collection tube samples were thawed overnight at 4 °C prior to processing. All samples were manually homogenised and weighed into separate aliquots for analysis. All samples and aliquots were kept on ice while processing and stored at −80 °C until thawed for analysis.

### Microbial organic acids, SCFA and pH analysis

Immediately after processing, faecal sample aliquots had three volumes of 1.68 mM heptanoic acid internal standard (IS) added per weight of faeces. Tubes were vortexed well, pH was measured and stored at −80 °C until analysis. When analysing microbial organic acid concentrations, aliquots were thawed on ice, vortexed well and centrifuged at 2095 xg for 5 minutes at 4 °C. Supernatants were transferred to microcentrifuge tubes which were centrifuged for a further 5 minutes at 15, 400 x g at 4 °C. 300 µL of supernatant was acidified with 10 µL 10% phosphoric acid and filtered with a Whatman PTFE 0.45 µm Mini-UniPrep tube. Filtrates were analysed via gas chromatography Agilent 6890 series.

### DNA extraction, library preparation and 16S rRNA gene sequencing

DNA was extracted from all samples using the Qiagen PowerMag Microbiome RNA/DNA Isolation kit following the manufacturer’s protocol. All samples were extracted and processed in duplicate. Extracted samples were checked for purity using the Nanodrop 2000 Spectrophotometer (ThermoFisher Scientific) and quantified using the Qubit Fluorometer (ThermoFisher Scientific). Based on these values the DNA was diluted and normalised to 5 ng/µL. Following normalisation, amplicon library preparation was performed using a modified dual-index library preparation approach amplifying the V4-V5 hypervariable regions of the 16S rRNA gene^18^. The first PCR (PCR 1) consisted of 12.5 µL KAPA HiFi (KAPA Biosystems – Roche), 5.8 µL 515 F primer, 5.8 µL 806 R primer^19^ and 1 µL of normalised DNA. PCR1 mix was amplified on the Thermocycler (BioRad) using the following program; (95 °C for 3 minutes, 98 °C for 20 seconds, 60 °C for 15 seconds, 72 °C for 30 seconds) x 25 cycles, 72 °C for 1 minute with a 10 °C hold time. PCR1 product size and quality was confirmed using a 1 K DNA chip on the Experion Automated Electrophoresis System (BioRad). Samples were then purified using AMPure beads (Beckman Coulter). A second subsequent PCR (PCR 2) consisted of 5 µL of cleaned PCR 1 product, 25 µL KAPA HiFi, 5 µL indexing primer i5, 5 µL indexing primer i7 and 10 µL sterile PCR-grade water. PCR2 mix was amplified on the Thermocycler using the following program; (95 °C for 3 minutes, 98 °C for 20 seconds, 65 °C for 15 seconds, 72 °C for 30 seconds) x 10 cycles, 72 °C for 1 minute and a 10 °C hold time. PCR2 product size and quality was confirmed using a 1 K DNA chip on the Experion Automated Electrophoresis System (BioRad). Samples were again purified using AMPure beads (Beckman Coulter).

PCR2 products were quantified using Qubit Fluorometric quantitation, and pooled in equimolar concentrations. The pooled sample was shipped to the Ramaciotti Centre for Genomics (University of New South Wales) where it was sequenced on one lane of an Illumina MiSeq instrument using a v2 reagent kit (500 cycle).

Raw 16S rRNA gene sequencing data were filtered, trimmed, merged and clustered into Operational Taxonomic Units (OTUs) at 97% similarity using a combination of proprietary^20^ and open-source (USEARCH) scripts^21^. Each OTU was classified in two ways; via the RDP Classifier and by finding the closest match in a set of curated reference sequences (RDP 16S Training Set + RefSeq. 16S). Any taxa that were not represented at over 1% relative abundance in at least one sample were removed.

### Statistical analysis

Microbial organic acids, SCFA and pH data were analysed using the non-parametric Wilcoxon Signed-Rank test. All statistical calculations on the microbiome data were conducted in R Studio using statistical packages made4 and VEGAN^22–24^. The Euclidean distance Principal Coordinates Analysis (PCoA) groupings were tested for significant separation using an adonis permutational test (9999 permutations). The Wilcoxon Signed-Rank test was performed to assess significant differences between taxonomy at the genus and phylum levels. A P-value of less than 0.05 was deemed significant after correcting for multiple comparisons using the False Discovery Rate (FDR) method.

## Results

### Participant recruitment, compliance and feedback

Out of the thirty participants recruited, four failed to complete their collections successfully, with two not collecting samples due to unrelated circumstances and two not following the sample collection instructions correctly which resulted in the fresh frozen sample being thawed when received at the laboratory. This compromised sample quality and these samples were excluded from the analyses. In total, twenty-six participants completed the study and supplied samples for analysis. The feedback received for the sampling process was positive and the majority of participants found it easy to complete a collection using the tubes. However, the amount of faecal sample transferred to the collection tubes varied widely amongst the participants with only five of the twenty-six participants successfully adhering to the manufacturer’s recommendations by transferring 2 grams or less to each tube. There was a large variation across the amounts of extra sample added to each tube. Seven participants added 0 to 1 gram extra to each tube, five added between 1 and 2 grams and nine participants added more than 2 grams extra to each tube.

### Bristol stool scale

Nineteen participants reported stool formation of types 3, 4 or 5. One participant reported type 2 (firm) and six participants reported type 6 or loose stool samples. No differences in study outcomes were observed when the data was stratified by the different faecal formation types, indicating that the collection methods provided the same preservation capacity across all faecal types.

### Microbial organic acid, SCFA and pH measurements

A significant difference was observed in pH measurements between the two sample collection methods (Table 1). Organic acid concentrations remained comparable except for acetic acid which was significantly different between the two collection methods (P = 0.002). There were no SCFAs detectable in the collection tube buffer. The acetic acid concentration differences were further analysed by stratifying the data into compliant and non-compliant sample groups (compliant, up to 2 grams faecal matter in collection tube; non-compliant, above 2 grams in collection tube). Acetic acid concentrations for non-complaint tube collections (n = 21) were significantly different (P = 0.011) to fresh frozen samples, however compliant collections (n = 5) were not significantly different to fresh frozen samples (Table 1).

**Table 1:** pH and SCFA mean ± SEM reported in µmol/gram wet weight faeces.

	Fresh Frozen	Collection tubes	P-value
pH	7.31 ± 0.06	7.42 ± 0.06	0.009*
Propionic acid	20.24 ± 2.16	19.22 ± 1.86	0.483
Isobutyric acid	2.47 ± 0.23	2.33 ± 0.22	0.434
Butyric acid	18.36 ± 1.83	17.58 ± 2.58	0.136
Isovaleric acid	3.84 ± 0.39	3.61 ± 0.40	0.561
Valeric acid	2.53 ± 0.14	2.65 ± 0.24	0.896
Caproic acid	1.25 ± 0.18	1.90 ± 0.47	0.288
Acetic acid	62.14 ± 5.37	54.33 ± 7.70	0.002*
Acetic acid – non-compliant (>2 grams faeces) (n = 21)	65.76 ± 6.12	57.45 ± 9.30	0.011*
Acetic acid – compliant (≤2 gram of faeces) (n = 5)	46.94 ± 9.00	41.23 ± 7.5	0.063

Significant differences between methods were determined with the Wilcoxon Signed Rank test after False Discovery Rate (FDR) correction (*P < 0.050).

### DNA quantification and microbiome profiling

**Figure 1:**
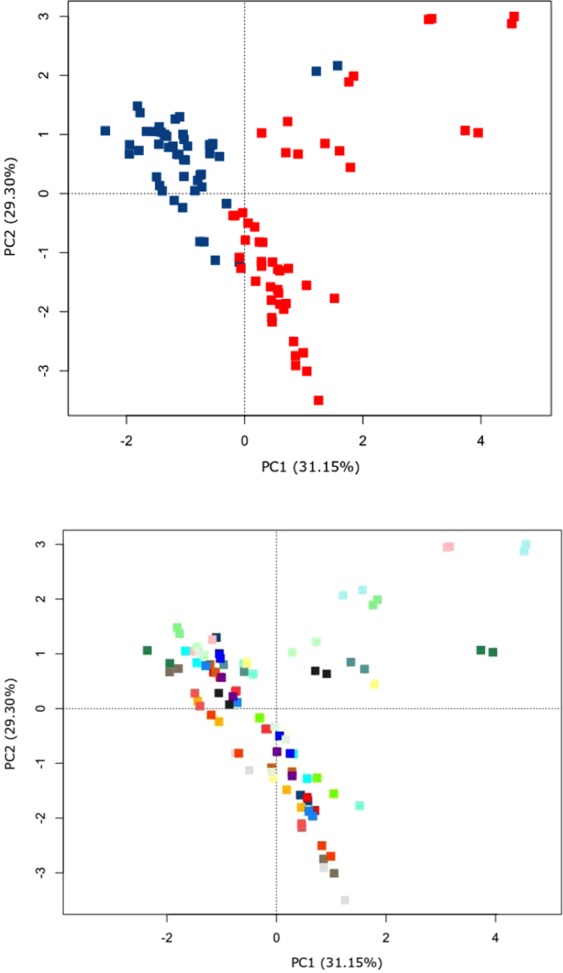
Principal Coordinates Analysis (PCoA) plot of the Euclidean distances of genus level relative abundance data. Data generated from MiSeq. 16S rRNA gene sequencing. 1 A: Fresh frozen samples (blue); collection tube samples (red) (P < 0.001, R^2^ = 0.21). 1B: Coloured by participants (n = 26) (P = 0.385, R^2^ = 0.01). Significance determined using the non-parametric adonis test (9999 permutations).

An average of 152,102 reads per sample were obtained across the samples sent for MiSeq. 16S rRNA gene sequencing. From a community perspective, a significant difference was evident between the fresh frozen and collection tube samples as shown in the Principal Coordinates Analysis (PCoA) plot of Euclidean distances (Fig. 1A) (P < 0.001, R^2^ = 0.21). No clustering was evident when comparing the differences between individual participant’s microbiome profiles as shown in Fig. 1B (P = 0.385, R^2^ = 0.01). Figure 1B also shows the two replicates per collection method being closely similar to each other, indicating robust technical replication within each collection method.

### Phylum-level profile

There was a clear difference between the community structures at phylum level based on collection method. Five out of eight measured phyla were significantly different between the two methods. Actinobacteria and Firmicutes were measured in higher abundance in the fresh frozen samples. Bacteroidetes, Lentisphaerae, and Proteobacteria, were measured in higher abundance in the collection tube samples. No differences were seen across Euryarchaeota, Synergistetes or Verrucomicrobia populations. No differences were found when the data was split into compliance groups, except for a significant increase in Euryarchaeota for non-compliant samples (Table 2).

**Table 2:** Relative microbial abundance (%) mean values ± SEM at phylum-level.

Phylum-level: fresh frozen vs tube collections			P-value
	Fresh frozen	Collection tubes	
Actinobacteria	11.42 ± 1.36	5.78 ± 0.81	0.001*
Bacteroidetes	7.7 ± 0.93	32.27 ± 1.56	<0.001*
Euryarchaeota	2.05 ± 0.52	0.7 ± 0.15	0.900
Firmicutes	77.63 ± 1.34	58.5 ± 1.43	<0.001*
Lentisphaerae	0 ± 0	0.1 ± 0.03	<0.001*
Proteobacteria	0.24 ± 0.04	1.46 ± 0.14	<0.001*
Synergistetes	0 ± 0	0.05 ± 0.03	0.860
Verrucomicrobia	0.91 ± 0.19	1.06 ± 0.21	1.000
Phylum-level: compliant (≤2 grams) (n = 21) vs non-compliant (>2 grams)(n = 5)			
	Non-compliant	Compliant	P-value
Actinobacteria	5.90 ± 0.90	5.10 ± 1.91	1.000
Bacteroidetes	31.07 ± 1.57	39.48 ± 5.00	0.260
Euryarchaeota	0.79 ± 0.17	0.13 ± 0.13	0.040*
Firmicutes	59.67 ± 1.44	51.47 ± 4.46	0.220
Lentisphaerae	0.06 ± 0.01	0.36 ± 0.15	0.120
Proteobacteria	1.51 ± 0.16	1.13 ± 0.22	0.880
Synergistetes	0.05 ± 0.03	0.01 ± 0.00	1.000
Verrucomicrobia	0.87 ± 0.20	2.21 ± 0.78	0.160

Data generated from MiSeq. 16S rRNA gene sequencing. Significant differences were determined with the Wilcoxon Signed Rank test after False Discovery Rate (FDR) correction (*P < 0.050 is considered significantly different).

### Genus-level profile

**Figure 2:**
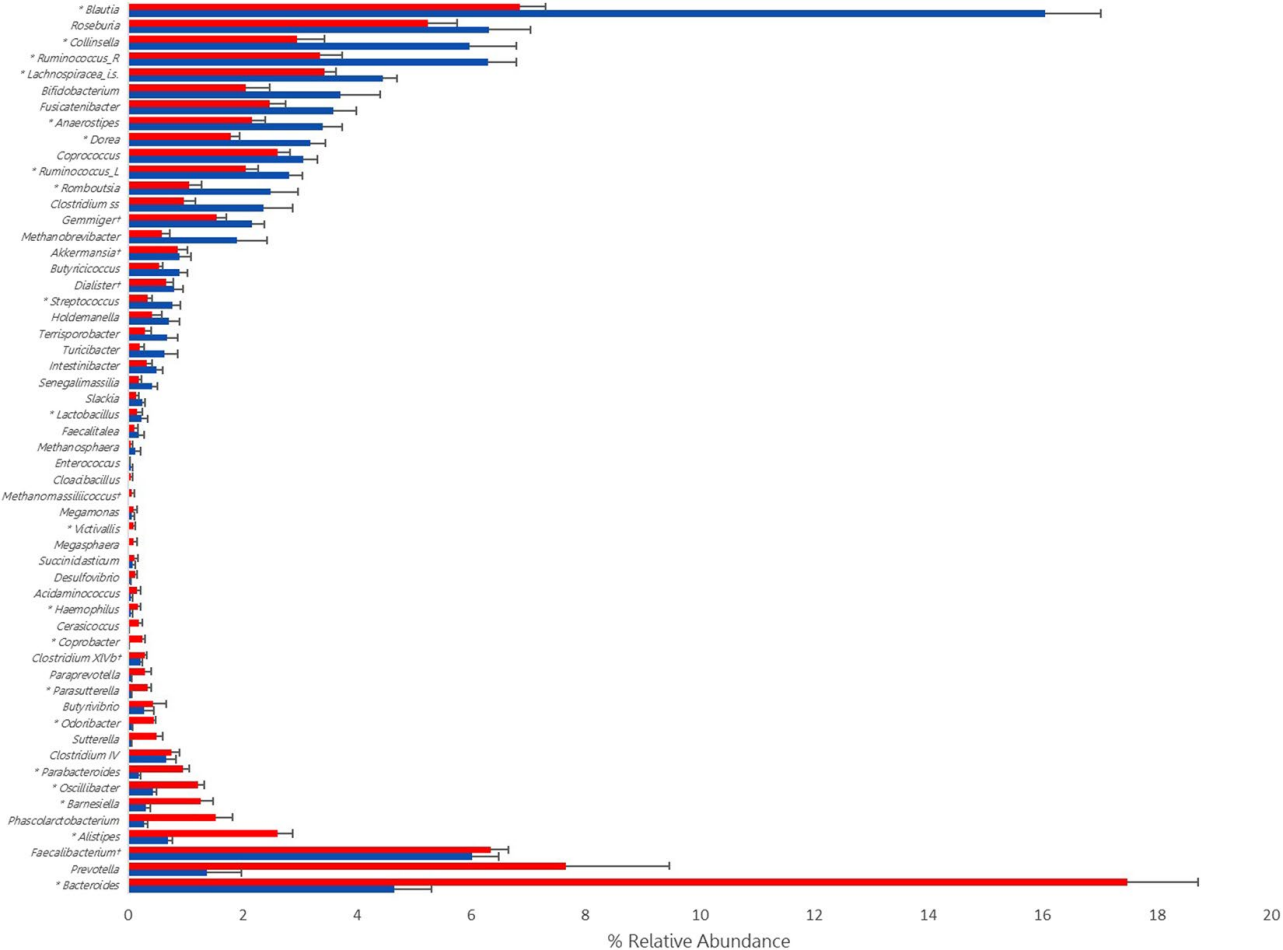
Bar chart displaying MiSeq. 16S rRNA gene sequencing (% relative abundance) data. Fresh frozen samples, blue bars; collection tube samples, red bars. Of the fifty-five taxa displayed the top twenty nine are all gram-positive and the bottom twenty six are all gram-negative with six exceptions denoted by^†^. Taxa with a preceding * were significantly different (P < 0.050) between the fresh frozen and collection tube samples. Ruminococcus_R - Ruminococcus within the Ruminococcaceae family; Ruminococcus_L - Ruminococcus within the Lachnospiraceae family; Lachnospiraceae_is - Lachnospiraceae incertae sedis. Clostridium_ss – Clostridium sensu stricto.

Fifty five bacterial genera were detected within the samples (>1% in at least one sample) (Fig. 2 and Supplementary Table 1). After scrutinizing these data a clear trend was apparent, where Gram-positive representatives were more abundant in the fresh frozen samples relative to collection tube samples. Conversely, Gram-negative bacteria were higher in the collection tubes compared to the fresh frozen samples. Only six taxa were exceptions to this trend (*Faecalibacterium*, *Clostridium* cluster *XIVb*, *Gemmiger*, *Dialister*, *Methanomassiliicoccus* and *Akkermansia*) where the greatest percent difference between the two methods was less than 0.63%.

**Figure 3:**
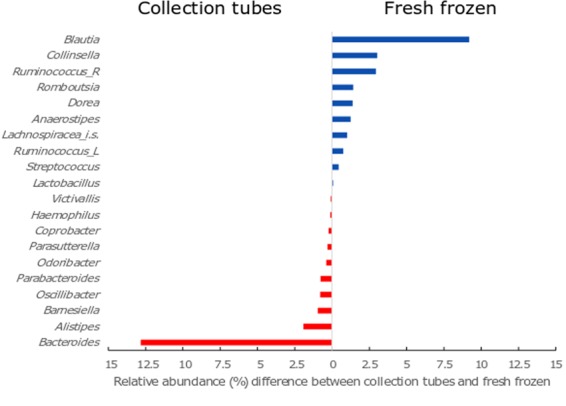
Twenty bacterial taxa that exhibited a statistically significant (P < 0.050) difference between % relative abundance in the frozen collection method compared to the tube collection method. The top ten genera (blue) are all gram-positive and at a relative higher abundance in the fresh frozen samples. The bottom ten genera (red) are all gram-negative and at a relative higher abundance in the collection tube samples. Ruminococcus_R - Ruminococcus with the Ruminococcaceae family; Ruminococcus_L - Ruminococcus with the Lachnospiraceae family; Lachnospiraceae_i.s.- Lachnospiraceae incertae sedis.

Of the fifty five taxa, twenty were significantly different between the two collection methods (Fig. 3). This was comprised of ten Gram-positive bacteria that were more abundant in the fresh frozen samples and ten Gram-negative bacteria which were more abundant in the collection tube samples.

Alpha diversity analysis was performed across all fresh frozen and collection tube samples (data not shown). No discernible difference was observed in the microbiome alpha diversity across the two methods.

## Discussion

This study has extended faecal sample handling knowledge in an important area of microbiome research. It has endorsed the possible use of collection tubes for faecal organic acid investigations and highlighted a trend which allows us to better understand collection method bias. For metabolomic applications it has been shown that effective methods are immediate transfer to 4 °C storage and extracting within one hour after collection^25^, immersion in 95% ethanol^26^, and using Cary-Blair faecal swabs which have been demonstrated to preserve content for metabolomics, metagenomics and transcriptomic applications^27^. Studies investigating the preservation of DNA support the use of faecal occult blood test (FOBT) cards^28^, faecal desiccation^29^ and RNA later^30^. The available literature reports a wide range of preferences for methodologies across these studies, with ambiguous results. For example, while one study supports the use of RNA later, others found that RNA later decreases the DNA yield, therefore impacting upon microbial population analysis^28,31^. However, despite incongruity between these studies, it is widely accepted that freezing (either at −20 °C or −80 °C) achieves optimal sample preservation^31^ and many studies use this approach as the best practice comparison when assessing the validity of other faecal preservation techniques.

Previous studies investigating sample storage, processing and preservation are often limited with test sample number, whereas samples from twenty-six participants, processed in duplicate, lends credence to our data. The design of our study was tailored to examine the use of collection tubes in a clinical trial setting. As such, participants were asked to send their tubes through the Australian postal system. Even though no tube samples persisted for more than six days in the post, it cannot be determined what environmental stresses the samples were exposed to during this time. Product specificities suggest that the tubes can be stored at room temperature for up to two years and still maintain quality DNA content. Therefore, it is assumed that the sample integrity should have been maintained under our test conditions.

Only five of the twenty six recruited participants were able to adhere to the collection guidelines and successfully transfer <2 grams of sample into the tubes, indicating that following the kit instructions was difficult. With 80% of participants adding varying degrees of excessive faecal sample this poses a substantial problem within a clinical trial setting where participants, with no prior sample handling training, are routinely required to collect samples in their own homes. Although ‘fill line’ marking on the tubes and manufacturer instructions are clear, this outcome indicates that further effort must be afforded in a clinical trial setting to ensure participants are provided adequate instruction on sampling requirements. The stated upper capacity of the tubes is 2 grams and it has been illustrated in this study that exceeding this limit may have implications on acetic acid metabolite measurements.

A recent review found that rapid freezing of faecal samples readily preserves the organic and SCFA content of a faecal sample^32^. While cryo-preservation is considered best practice for SCFA, it can be complex and costly to implement across large clinical trials utilising field collections. This study has investigated the capacity of commercially available stool preservation tubes to also preserve faecal SCFA metabolites. Results demonstrate a significant difference in acetic acid concentrations between the fresh frozen and collection tube samples. However, when grouped into complaint vs non-compliant samples it was evident that the 2-gram capacity of the tube is an important factor for determining acetic acid content. Unfortunately, with only five compliant subjects in the present study, this matter requires greater sample numbers to clearly define the impact of sample overloading on SCFAs. A previous review of faecal collection methods suggested that ambient temperature collection tubes were unsuitable for metabolic content preservation^27^. However, the current study has shown that the Stool Nucleic Acid Collection and Preservation Tubes, when used to manufacturers specifications, may be suitable for faecal sample SCFA preservation.

Our objectives were to confirm the microbiome preservation capacity of stool collection tubes and investigate their suitability for preserving SCFA metabolites. Unexpectedly this study has illustrated a distinct shift in microbial community structure that is linked with cellular wall/membrane structure. At phylum and genus level the data suggested that the collection methods significantly bias microbiome profiles. It was apparent that the fresh frozen samples favoured an increased proportion of Gram-positive representatives and a decreased proportion of Gram-negative genera. Conversely, the collection tubes had a lower proportion of Gram-positive genera and a relative higher abundance of Gram-negative genera. Previous studies have theorised a freezing bias being attributed to the cellular wall structure of the microbial population^33,34^ but not illustrated the clear trend evident in our study. Furthermore, several other studies have reported results that are consistent with the collection method bias illustrated in our study. For example, a recent investigation reported a decrease in the Gram-positive Actinobacteria phylum in OMNIgene.GUT tubes when compared to frozen samples^35^. Another study reported an increase in Gram-negative *Sutterella* in the OMNIgene.GUT tube when compared to fresh frozen^7^. Although not significant, our results follow the same trend for the *Sutterella* genus. An additional study investigated the OMNIgene.GUT preservation tube in a clinical setting which highlighted differences at phylum level but attributed it to the method used for DNA extraction. However, their results are congruent with ours in that the Bacteroidetes were measured in higher abundance in the collection tubes and the Actinobacteria were measured in lower abundance in the collection tubes^36^. Many studies report that a difference in individual microbiome profiles is the principal driver of beta diversity variation, exceeding differences caused by collection method, storage conditions or nucleic acid extraction techniques^28,34,35^. However, we have demonstrated that collection method is the principal driver of variation between samples in our study.

Further investigations into the effects of freezing with and without preservation buffer are necessary and it is not within the scope of this project to describe a potential mechanism that is generating the Gram status bias seen across the two different collection methods. Our results indicate that it may be dictated by bacterial cell wall/membrane structure however, it is unclear whether the bias was due to a sensitivity of Gram-negative bacteria to freezing or whether the preservative buffer in the collection tubes is adversely affecting Gram-positive bacteria. The exact composition of these preservation solutions are proprietary, but likely contain anionic detergents, buffers and/or salts that are not only bacteriostatic, but potentially bactericidal, to arrest any further microbial growth^33^. Bacterial cell wall structure may also influence DNA extraction efficiency as shown in a study where a higher abundance of Gram-positive genera were detected in samples stored under frozen conditions^33^. In order to better understand the collection method bias observed in the current study, each method could be compared with freshly voided and extracted faecal samples, prior to freezing. This will help determine whether the source of the bias is due to freezing, preservation buffer or a combination of the two.

It remains clear that all preservation methods, even those considered to be gold standard such as cryo-preservation, present a level of sample collection bias. This study has illustrated a clear bias in faecal collection methods that sets us on a path to define this trend in order to deliver more robust, reproducible data on the gut microbiome and links to health. Future studies that utilise faecal collections should carefully consider the most appropriate method to minimise the potential to introduce microbial profile bias.

## Supplementary information

Microbial relative abundance (%) at genus-level
