# Supplementary material for: Human faecal collection methods demonstrate a bias in microbiome composition by cell wall structure
Source: Sci Rep. 2019 Nov 14;9:16831. doi: 10.1038/s41598-019-53183-5 (PMC6856092; doi:10.1038/s41598-019-53183-5)
Supplement: Supplementary file 1 — Microbial relative abundance (%) at genus-level [file 41598_2019_53183_MOESM1_ESM.pdf]

Title: Human faecal collection methods demonstrate a bias in microbiome composition by cell wall structure**.**

Authors:

Emma-Jane Watson[1], Jennifer Giles[1], Benjamin L. Scherer[1] and Dr Paul Blatchford[2,*].

[1] CSIRO Health and Biosecurity, Gate 13 Kintore Avenue, Adelaide, South Australia 5000, Australia

[2] Zespri International Limited, 400 Maunganui Road, Mt Maunganui 3149, New Zealand.

[*] paul.blatchford@zespri.com

	**Mean Bacterial Abundance (%) ±SEM**				
	**Fresh frozen**	**Collection tube**	**Gram Status**	**Percent Difference**	**P-Value**
Bacteroides	4.66 ± 0.65	17.48 ± 1.25	Negative	-12.82	<0.001*
Prevotella	1.37 ± 0.61	7.66 ± 1.81	Negative	-6.29	0.192
Alistipes	0.69 ± 0.08	2.61 ± 0.26	Negative	-1.92	<0.001*
Phascolarctobacterium	0.28 ± 0.06	1.53 ± 0.3	Negative	-1.25	0.520
Barnesiella	0.31 ± 0.07	1.27 ± 0.21	Negative	-0.96	<0.010*
Oscillibacter	0.43 ± 0.06	1.22 ± 0.11	Negative	-0.79	<0.001*
Parabacteroides	0.19 ± 0.03	0.96 ± 0.11	Negative	-0.77	<0.001*
Sutterella	0.04 ± 0.02	0.5 ± 0.11	Negative	-0.46	0.048*
Odoribacter	0.06 ± 0.01	0.45 ± 0.03	Negative	-0.39	<0.001*
Faecalibacterium†	6.02 ± 0.46	6.34 ± 0.32	Positive	-0.32	1.000
Parasutterella	0.04 ± 0.01	0.34 ± 0.06	Negative	-0.3	<0.001*
Paraprevotella	0.04 ± 0.02	0.29 ± 0.11	Negative	-0.25	1.000
Coprobacter	0.02 ± 0	0.25 ± 0.05	Negative	-0.23	<0.001*
Cerasicoccus	0.01 ± 1	0.19 ± 0.06	Negative	-0.18	0.280
Butyrivibrio	0.28 ± 0.16	0.43 ± 0.24	Negative	-0.15	0.420
Haemophilus	0.05 ± 0.02	0.17 ± 0.05	Negative	-0.12	0.006*

Acidaminococcus	0.05 ± 0.03	0.15 ± 0.06	Negative	-0.1	0.280
Desulfovibrio	0.03 ± 0.01	0.12 ± 0.04	Negative	-0.09	0.680
Megasphaera	0 ± 0	0.09 ± 0.06	Negative	-0.09	0.400
Victivallis	0 ± 0	0.09 ± 0.03	Negative	-0.09	<0.001*
Clostridium IV	0.67 ± 0.17	0.76 ± 0.13	Variable	-0.09	0.680
Clostridium XIVb†	0.21 ± 0.03	0.29 ± 0.04	Positive	-0.08	0.052
Methanomassiliicoccus†	0 ± 0	0.06 ± 0.04	Positive	-0.06	0.520
Cloacibacillus	0 ± 0	0.04 ± 0.03	Negative	-0.04	0.660
Megamonas	0.06 ± 0.05	0.09 ± 0.06	Negative	-0.03	0.110
Succiniclasticum	0.07 ± 0.05	0.1 ± 0.07	Negative	-0.03	1.000
Akkermansia†	0.9 ± 0.19	0.87 ± 0.17	Negative	0.03	1.000
Enterococcus	0.05 ± 0.03	0.02 ± 0.01	Positive	0.03	1.000
Lactobacillus	0.23 ± 0.11	0.16 ± 0.09	Positive	0.07	0.003*
Faecalitalea	0.19 ± 0.09	0.11 ± 0.06	Positive	0.08	0.660
Methanosphaera	0.13 ± 0.09	0.05 ± 0.03	Positive	0.08	0.600
Slackia	0.24 ± 0.06	0.14 ± 0.04	Positive	0.1	0.400
Dialister†	0.8 ± 0.16	0.66 ± 0.13	Negative	0.14	0.880
Intestinibacter	0.5 ± 0.1	0.33 ± 0.08	Positive	0.17	0.380
Senegalimassilia	0.42 ± 0.09	0.18 ± 0.05	Positive	0.24	0.600
Holdemanella	0.71 ± 0.19	0.42 ± 0.16	Positive	0.29	1.000
Butyricicoccus	0.89 ± 0.14	0.54 ± 0.06	Positive	0.35	0.360
Terrisporobacter	0.68 ± 0.18	0.3 ± 0.1	Positive	0.38	0.360
Streptococcus	0.78 ± 0.13	0.34 ± 0.07	Positive	0.44	0.002*
Teuricibacter	0.64 ± 0.22	0.2 ± 0.08	Positive	0.44	0.150
Coprococcus	3.07 ± 0.24	2.61 ± 0.22	Positive	0.46	0.240
Gemmiger†	2.16 ± 0.22	1.54 ± 0.18	Negative	0.62	0.086
Ruminococcus_L	2.18 ± 0.24	2.05 ± 0.22	Positive	0.76	0.30*
Lachnospiracea_is	4.45 ± 0.26	3.43 ± 0.2	Positive	1.02	0.019*
Roseburia	6.32 ± 0.72	5.25 ± 0.51	Positive	1.07	1.000
Fusicatenibacter	3.59 ± 0.4	2.47 ± 0.28	Positive	1.12	0.118
Anaerostipes	3.4 ± 0.35	2.16 ± 0.23	Positive	1.24	0.009*
Methanobrevibacter	1.91 ± 0.52	0.58 ± 0.14	Positive	1.33	1.000
Clostridium_ss	2.36 ± 0.51	0.98 ± 0.2	Positive	1.38	0.066
Dorea	3.18 ± 0.27	1.8 ± 0.15	Positive	1.38	<0.001*
Romboutsia	2.49 ± 0.48	1.07 ± 0.21	Positive	1.42	0.005*
Bifidobacterium	3.72 ± 0.69	2.06 ± 0.42	Positive	1.66	0.076
Ruminococcus_R	6.3 ± 0.5	3.36 ± 0.38	Positive	2.94	<0.001*
Collinsella	5.98 ± 0.82	2.95 ± 0.48	Positive	3.03	0.010*
Blautia	16.05 ± 0.98	6.85 ± 0.45	Positive	9.2	<0.001*

Supplementary Table 1: Microbial relative abundance (%) at genus-level.

Fifty-five bacterial genera in fresh frozen samples compared to DNA collection tube samples with associated Gram stain classification. There are six exceptions to the cellular wall

structure trend, denoted by **†**. *Significant differences between methods were determined with the Wilcoxon Signed Rank test after False Discovery Rate (FDR) correction (P*<0.050).*

Ruminococcus R – Ruminococcus within the Ruminococcaceae family; Ruminococcus L – Ruminococcus within the Lachnospiraceae family; Lachnospiraceae IS – Lachnospiraceae incertae sedis.
